# Axonin-1/TAG-1 is required for pathfinding of granule cell axons in the developing cerebellum

**DOI:** 10.1186/1749-8104-3-7

**Published:** 2008-03-17

**Authors:** Thomas Baeriswyl, Esther T Stoeckli

**Affiliations:** 1 Institute of Zoology, University of Zurich, Winterthurerstrasse, 8057 Zurich, Switzerland

## Abstract

**Background:**

Neural development consists of a series of steps, including neurogenesis, patterning, cell migration, axon guidance, and finally, synaptogenesis. Because all these steps proceed in a constantly changing environment, functional gene analyses during development have to take time into account. This is quite challenging, however, as loss-of-function approaches based on classic genetic tools do not allow for the precise temporal control that is required for developmental studies. Gene silencing by RNA interference (RNAi) in combination with the chicken embryo or with cultured embryos opens new possibilities for functional gene analysis *in vivo*. Axonin-1/TAG-1 is a cell adhesion molecule of the immunoglobulin superfamily with a well defined temporal and spatial expression pattern in the developing vertebrate nervous system. Axonin-1/TAG-1 was shown to promote neurite outgrowth *in vitro *and to be required for commissural and sensory axon pathfinding *in vivo*.

**Results:**

To knock down axonin-1 in a temporally and spatially controlled manner during development of the nervous system, we have combined RNAi with the accessibility of the chicken embryo even at late stages of development. Using *ex ovo *RNAi, we analyzed the function of axonin-1/TAG-1 in cerebellar development. Axonin-1 is expressed in postmitotic granule cells while they extend their processes, the parallel fibers. In the absence of axonin-1 these processes still extend but no longer in a parallel manner to each other or to the pial surface of the cerebellum.

**Conclusion:**

Axonin-1/TAG-1 is required for the navigation, but not for the elongation, of granule cell processes in the developing cerebellum *in vivo*.

## Background

Axonin-1 (AX-1)/TAG-1 is a cell adhesion molecule of the immunoglobulin superfamily that was shown to be an axon guidance cue in the central nervous system *in vivo *[1,2]. Commissural axons in the spinal cord require AX-1 for midline crossing [3]. In the absence of interactions between growth cone AX-1 and floor-plate NrCAM the floor plate is perceived as repulsive and growth cones fail to enter [4]. Sensory neurons from dorsal root ganglia depend on AX-1 for subpopulation-specific navigation to the gray matter of the spinal cord [5]. In the absence of AX-1 function, nociceptive fibers failed to innervate their target layers in the dorsal spinal cord and extended into areas normally innervated by mechanoreceptive fibers. In the cerebellum, AX-1 is expressed in postmitotic granule cells at the time when they extend their processes, the parallel fibers [6-8].

The cerebellum is responsible for motor coordination but is also involved in cognitive processes [9,10]. Malformations or damage to the cerebellum have been linked to several human disabilities, including ataxia, cerebral palsy, and epilepsy [9]. In line with the more recent literature describing a contribution of the cerebellum to cognitive processes, changes in cerebellar structure and function have been linked to mental retardation, autism, and schizophrenia [10]. The development of the cerebellum has been studied in mouse [9,11-14] and in chicken embryos [15-19]. Cells of the cerebellum originate from rhombomere 1, the anterior-most part of the hindbrain [19]. With one notable exception, the granule cells, all cells are born in the ventricular zone and migrate radially to reach their final destination in the mature cerebellum [12,14]. The rhombic lip, the contact site between the roof plate of the fourth ventricle and the neural tube, gives rise to precursors of granule cells. These migrate tangentially on the pial surface of the cerebellar anlage to form the external germinal layer (EGL) [19-22]. After proliferation in the outer EGL, granule cell precursors differentiate in the inner EGL and extend their axons, the parallel fibers. Together, parallel fibers and dendrites of Purkinje cells form the molecular layer (ML). After the initial formation of parallel fibers, granule cells migrate radially through the Purkinje cell layer to form the inner granule cell layer [12]. Thus, the only cell bodies that remain in the ML are interneurons, stellate and basket cells that originate from the ventricular zone [23,24]. Although these processes are known at the cellular level, the molecular mechanisms regulating these distinct steps are poorly understood.

To test for a role of AX-1 in parallel fiber extension in the developing cerebellum, we used *ex ovo *RNA interference (RNAi), a combination of RNAi and *ex ovo *culturing of chicken embryos. In contrast to our previously established method, *in ovo *RNAi [25], *ex ovo *RNAi enhances the accessibility of chicken embryos for loss-of-function approaches at late stages of development.

We developed *in ovo *RNAi as a tool to study gene function in a temporally controlled manner during development of the chicken nervous system [25-27]. Using this technique, we demonstrated, for instance, additional functions of the morphogen Sonic hedgehog (Shh) beyond patterning and differentiation [28]. During early stages of development, Shh acts as a ventralizing factor in spinal cord patterning [29]. Later, still using Patched and Smoothened to mediate its effect, Shh attracts commissural axons toward the floor plate [30]. Only a few hours later, Shh switches receptors and, mediated by Hip (Hedgehog-interacting protein), acts as a guidance cue for postcommissural axons [31]. The analysis of Shh's function clearly demonstrates the importance of tight temporal control of gene silencing during embryonic development [26,27].

*Ex ovo *RNAi extends the usefulness of the chicken embryo as a model organism to study later stages of neural development during which the embryo is no longer easily accessible in the egg. Chicken embryos can be transferred from the egg to a plastic dish without detrimental effects on their development. The absence of the eggshell enhances access to the developing embryo for experimental manipulation. *Ex ovo *RNAi allowed us to study the role of AX-1 in parallel fiber formation. In the absence of AX-1 function, neurites of granule cells still extended but failed to navigate correctly in the developing ML. Rather than extending in a parallel manner, they invaded the layer of proliferating granule cells at the pial surface of the cerebellum.

## Results

### Embryos grown *ex ovo *can easily be accessed for manipulation of gene expression in a temporally and spatially controlled manner

To enhance its accessibility during later stages of development, the chicken embryo can be cultured in a dish without adverse effects on development [32-35]. To have access to the developing cerebellum, we transferred embryos to a domed dish after the second day of incubation (Additional file 1). As described previously [35], the transfer should be done no later than embryonic day 2.5 (E2.5) for best survival of the embryo. The survival rate of embryos depended on several factors, including temperature and humidity in the incubator, as well as the time of embryo transfer. Routinely, we found 62% of the embryos alive on E8, the time of injection in this study. At that time, embryos grown in a dish did not differ from embryos kept in the egg. Similarly, the comparison of cerebellar development between embryos developing in the egg and those developing in a dish for up to ten days after transfer (the latest time point that we analyzed) revealed no differences (Additional file 2).

**Figure 1:**
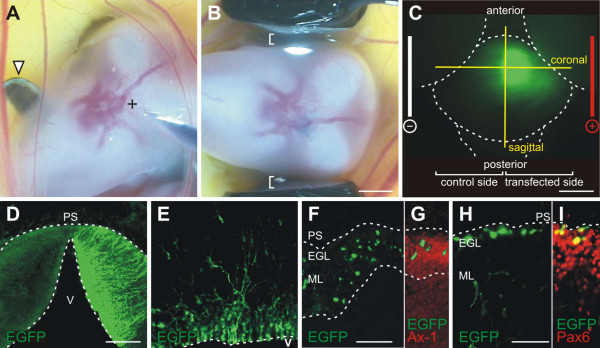
**Different cerebellar layers can be targeted by *ex ovo *electroporation.** The cerebellar anlage is positioned right under the bifurcation of the middorsal sinus and the middle cerebral vein. **(a) **The injection site is indicated by +. The head of the embryo was turned and stabilized with a hook prepared from a spatula (arrowhead). **(b) **Tweezer electrodes were placed parallel to the head. It was important to avoid contact between embryo and electrode (indicated by white brackets) to prevent tissue damage. **(c,d) **One half of the cerebellum was successfully transfected by electroporation. The untransfected half of the cerebellum could serve as an internal control for the analysis of phenotypes. (d) Successful transfection of one half of the cerebellum after *ex ovo *electroporation with platelet electrodes is shown in a 250-μm-thick coronal slice. **(e-i) **Depending on the depth of the injection, different cerebellar layers could be targeted: deep injections into the ventricle resulted predominantly in transfection of the ventricular zone labeling all cell types proliferating and migrating from there, such as Purkinje cells, interneurons, and glia cells (e). With superficial injections the developing ML (f,g) and granule cells of the external germinal layer (h,i) were transfected. Parallel fibers in the developing ML were visualized using AX-1 as a marker (g). Granule cells were identified by Pax6 (i). EGL, external germinal layer; ML, molecular layer; PS, pial surface; V, ventricle. Bar: 2 mm in (a,b), 1 mm in (c), 500 μm in (d), 100 μm in (e-g), 50 μm in (h,i).

Injections into the cerebellum had to be carried out without direct visual control. However, the blood vessels that are readily visible through the skin could be used as landmarks (Figure 1a). By using an expression plasmid encoding enhanced green fluorescent protein (EGFP), the proper injection site, the required depth of the injection, as well as the best electroporation settings were determined. For electroporations we placed the head of the embryo between tweezer electrodes. To prevent tissue damage, direct contact between embryo and electrodes was carefully avoided (Figure 1b). The cerebellum was efficiently transfected with 6 pulses of 99 ms duration at 40 V, as judged by the number of EGFP-expressing cells (Figure 1d–i). Only one half of the cerebellum expressed the transgene, because the negatively charged nucleic acids were migrating towards the anode (Figure 1c,d). Therefore, the untransfected side of the cerebellum could be used as a control for the analysis of potential phenotypes. When injections were carried out at Hamburger and Hamilton stage 34 (HH34; E8) [36], the survival rate one day after the experiment was 72%. However, the time point of the injection is not restricted to HH34. Injections and electroporations were performed successfully until HH36 (E10; not shown).

Depending on the depth of the injection, different cerebellar layers could be targeted. Injections into the ventricle resulted in transfection of the ventricular zone, the origin of Purkinje cells and interneurons (Figure 1e). After superficial injections into the cerebellar anlage, cells of the developing ML (Figure 1f,g) and granule cells in the EGL were transfected (Figure 1h,i), similar to observations made in postnatal rat pups [37]. To get efficient transfection of all cerebellar layers, the glass capillary was inserted into the ventricle and the injection pressure was maintained during its retraction. For this study, we used only sections that were successfully transfected in the EGL and the developing ML. For analyses of experimental and control embryos, we carefully matched cerebellar sections with respect to their anteroposterior and mediolateral positions.

### *Ex ovo *RNAi does not induce apoptosis or morphological changes

**Figure 2:**
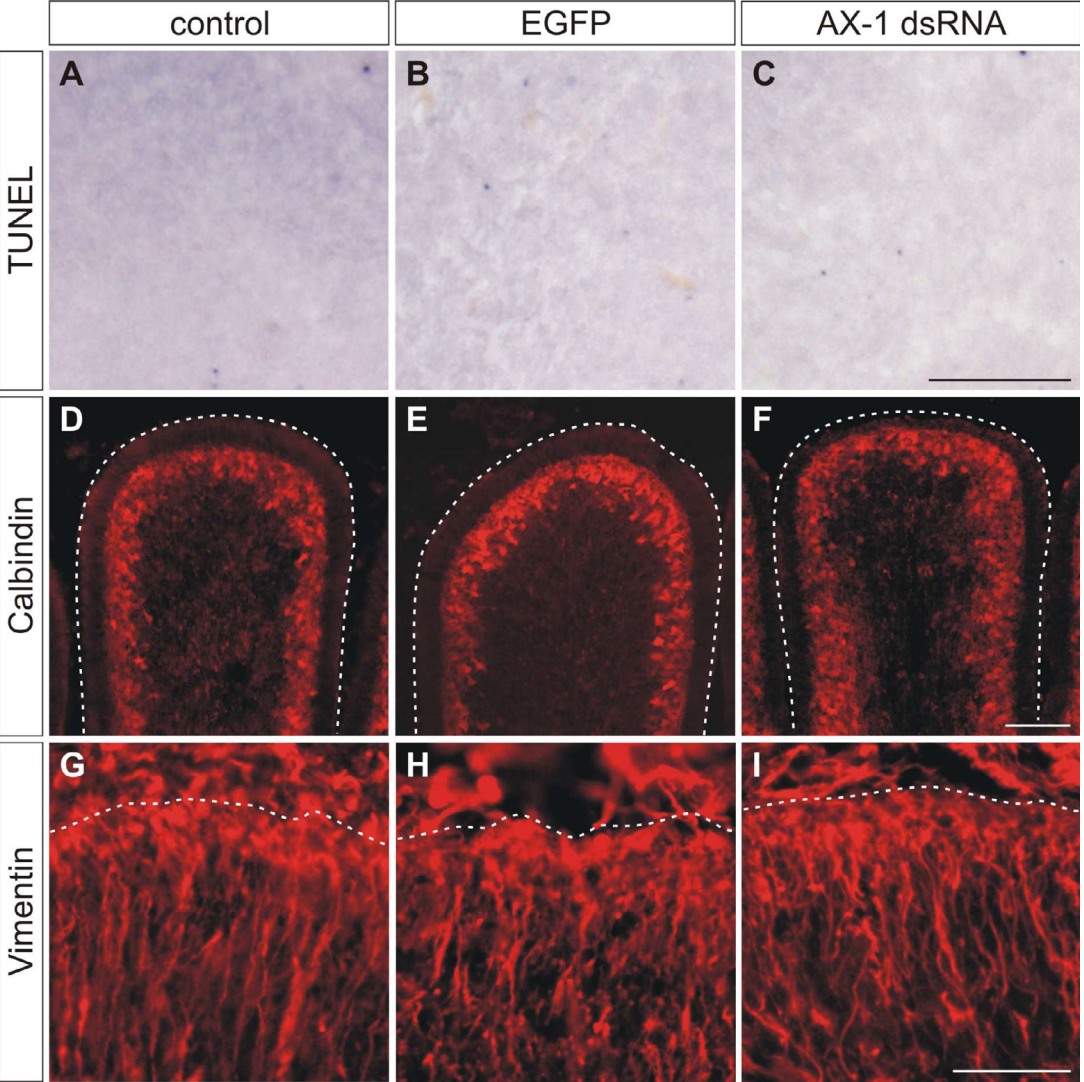
**Ex ovo electroporation and RNAi using long dsRNA does not induce apoptosis and does not disturb cerebellar organization.(a-c) **Apoptosis was investigated by TUNEL in 30-μm-thick sagittal sections at HH35, one day after electroporation. Corresponding areas are shown in (a-c). No difference in apoptosis was detected between untreated embryos (a), embryos injected with EGFP plasmids alone (b), and embryos injected with AX-1 dsRNA (c). **(d-f) **The organization and the development of Purkinje cells analyzed by Calbindin staining at HH38 did not differ between untreated control embryos (d), EGFP-expressing control embryos (e), and embryos electroporated with AX-1 dsRNA (f). **(g-i) **Similarly, no changes in Bergmann glia cells were detectable at HH37 after staining for Vimentin, when untreated (g), EGFP-expressing control embryos (h), and embryos lacking AX-1 (i) were compared. Bar: 100 μm in (a-c), 200 μm in (d-f), and 50 μm in (g-i).

To make sure that injection and subsequent electroporation of plasmids encoding EGFP or of long double-stranded RNA (dsRNA) into the cerebellum did not induce non-specific effects, we checked for apoptosis and histological changes in the transfected area of the cerebellum (Figure 2). Apoptosis was compared between untreated control embryos (Figure 2a), control embryos injected and electroporated with the EGFP plasmid alone (Figure 2b), and experimental embryos injected and electroporated with EGFP and dsRNA derived from AX-1 (Figure 2c). We did not find any difference in apoptosis between control and experimental embryos, indicating that our experimental procedure did not induce cell death. This is consistent with our previous findings demonstrating that injection and electroporation of long dsRNA did not induce unspecific effects in the chicken spinal cord [25].

The comparison of sections from control-injected/electroporated and untreated control embryos stained with methylene blue confirmed the absence of morphological changes in experimental embryos (not shown). The cerebellar foliation and the organization of the EGL were unchanged three days after electroporation. Furthermore, the organization of Purkinje cells and Bergmann glia cells was visualized by staining for Calbindin (HH38; Figure 2d–f) and Vimentin (HH37; Figure 2g–i), respectively. No changes were observed when experimental embryos were compared with control embryos three and four days after electroporation, respectively. Taken together, we did not find any evidence that *ex ovo *RNAi induced apoptosis, morphological changes, or developmental delays during cerebellar development.

### *Ex ovo *RNAi effectively silences the targeted gene

**Figure 3:**
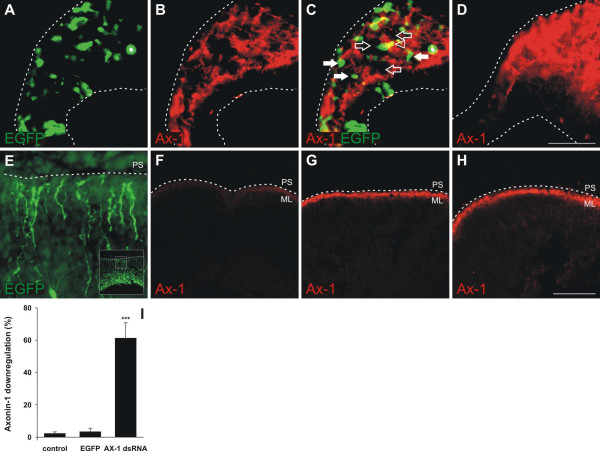
**Gene silencing by *ex ovo *RNAi is efficient.** The efficiency of AX-1 downregulation was demonstrated in caudal sections taken from HH35 cerebella one day after injection and electroporation of dsRNA derived from AX-1 mixed with a plasmid encoding EGFP (50:1). **(a) **EGFP expression was used to identify the electroporated area of the cerebellum. **(b,c) **Expression of AX-1 was lost from many cells after *ex ovo *RNAi, resulting in a patchy appearance of the granule cell layer (b; c, open arrows). **(d) **In corresponding sections of a control embryo AX-1 staining appeared homogenous. (c) The number of EGFP-expressing cells (white arrows) was much smaller than the number of cells that failed to express AX-1 due to the molecular ratio of 50:1 for dsRNA and EGFP plasmid. Very few cells were yellow, indicating both EGFP and AX-1 expression (c, arrowhead). For quantitative analysis, 30-μm-thick sagittal sections were stained for AX-1. **(e) **EGFP expression was used to identify the electroporated area of the cerebellum. Staining intensities were compared between experimental (e,f) and control (EGFP) embryos (h) at HH35, one day after electroporation. **(f,i) **On average, the injection and electroporation of AX-1 dsRNA reduced the AX-1 protein level in the transfected area by 61.3 ± 9.4% (n = 4 embryos; ***P < 0.0001 for comparison with both control groups). **(g) **As expected, there was no change in AX-1 protein levels in the untransfected part of the cerebellum of embryos treated with dsRNA. **(h,i) **AX-1 expression in transfected areas of embryos treated with the EGFP plasmid alone did not differ from untreated control embryos (not shown; i). The ratio of AX-1 staining was 3.4 ± 2.2% (n = 5) for EGFP-control embryos and 2.3 ± 0.9% (n = 4) in untreated control embryos. The insert in (e) shows a low magnification image of the section. The box corresponds to the high magnification image shown in (e). PS, pial surface. Bar: 50 μm in (a-d), 200 μm in (e-h).

AX-1 is expressed in postmitotic granule cells in the EGL of the developing cerebellum ([6-8]; this study). To demonstrate efficient downregulation of AX-1 in these cells by *ex ovo *RNAi, we mixed dsRNA derived from AX-1 with a plasmid encoding EGFP at a molecular ratio of 50:1. In all experiments EGFP expression was used to identify the position and the size of the electroporated area in the cerebellum. Electroporation of AX-1 dsRNA efficiently knocked down AX-1 protein (Figure 3). Caudal cerebellar sections taken from an experimental embryo exhibited a patchy pattern of AX-1 expression (Figure 3b) in contrast to the homogenous appearance in a control embryo (Figure 3d). As expected, the vast majority of EGFP-expressing cells did not express AX-1 (Figure 3c). Consistent with the 50-fold excess of dsRNA molecules compared to the EGFP plasmid, we found many cells that no longer expressed AX-1 but failed to express EGFP.

For the quantification of gene silencing we calculated the relative intensity of AX-1 staining in the electroporated area (Figure 3f) compared to the corresponding non-targeted area of the cerebellum (Figure 3g). A strong decrease of more than 60% of the AX-1 staining intensity was seen in the electroporated area of the cerebellum (identified by EGFP expression; Figure 3e) when embryos were injected and electroporated with AX-1 dsRNA (Figure 3i). As expected, there was no downregulation of AX-1 in the transfected area of embryos injected with the EGFP plasmid alone (Figure 3h,i).

**Figure 4:**
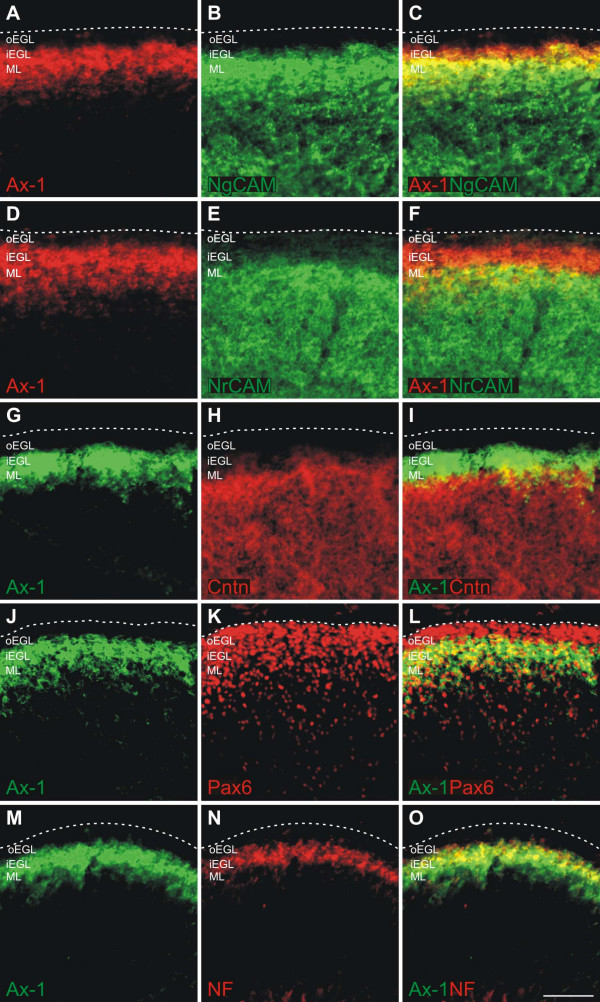
**AX-1 expression in postmitotic granule cells precedes expression of NgCAM, NrCAM, and Contactin/F11.(a,d,g,j,m) **AX-1 is a marker for postmitotic granule cells that start to extend processes in the inner EGL [6,8]. **(a-c) **Double-staining of sagittal sections taken from HH35 brains demonstrated that AX-1 (a) labeled young postmitotic granule cells in the inner EGL (iEGL) that were not yet NgCAM-positive (b; merged in c). Therefore, an overlap between AX-1 and NgCAM was only found in the ML, where older granule cells are located (c). **(d-f) **Double staining for AX-1 (d) and NrCAM (e) revealed that there was less overlap between AX-1 and NrCAM (f) compared to AX-1 and NgCAM (c). **(g-i) **Similarly, there was little overlap (i) between AX-1 (g) and Contactin/F11 (h), consistent with previous reports [39]. **(j-l) **AX-1 is expressed in postmitotic granule cells but not in granule cell precursors in the outer EGL (oEGL) that are still proliferating (j). Precursors and postmitotic granule cells are identified by Pax6 staining (k) [42]. The overlap between AX-1 and Pax6 is restricted to the inner EGL (l). **(m-o) **AX-1 reactivity (m) mostly overlapped with RMO270 reactivity (n), demonstrating that the vast majority of the neurofilament signal at HH35 was generated by granule cells (o). Bar: 50 μm.

**Figure 5:**
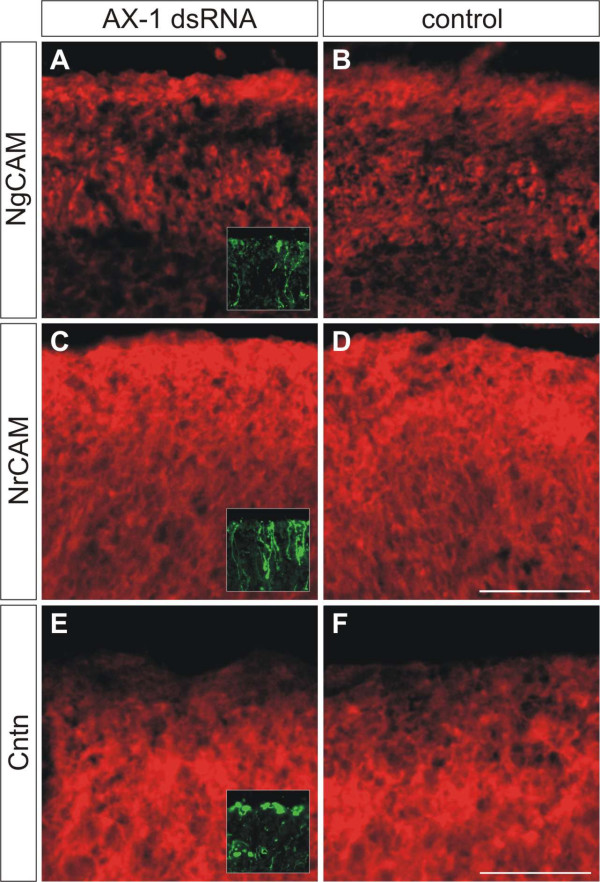
**Downregulation of AX-1 does not change the expression of related cell adhesion molecules.** We compared expression of related immunoglobulin superfamily cell adhesion molecules between **(a,c,e) **embryos lacking AX-1 and **(b,d,f) **age-matched untreated control embryos at HH35, one day after *ex ovo *RNAi. We found no changes in NgCAM (a,b), NrCAM (c,d), and Contactin/F11 expression (e,f). Inserts in (a,c,e) show adjacent sections stained for EGFP to demonstrate that sections were taken from the electroporated area of the cerebellum. Bar: 100 μm in (a-d), 50 μm in (e,f).

In order to demonstrate the specificity of *ex ovo *RNAi, we monitored the expression of the related cell adhesion molecules NgCAM, NrCAM, and Contactin/F11 that are expressed in the developing chicken cerebellum (Figure 4). In agreement with published reports, we found that AX-1 preceded NgCAM, NrCAM, and Contactin/F11 expression in postmitotic granule cells [38-40]. After downregulation of AX-1 no changes in the expression of NgCAM, NrCAM, and Contactin/F11 were observed (Figure 5). Taken together, these results indicate that *ex ovo *RNAi with long dsRNA derived from AX-1 effectively knocked down AX-1 levels without affecting non-targeted but related genes expressed in the cerebellum.

### Downregulation of AX-1 by *ex ovo *RNAi results in aberrant extension of parallel fibers

**Figure 6:**
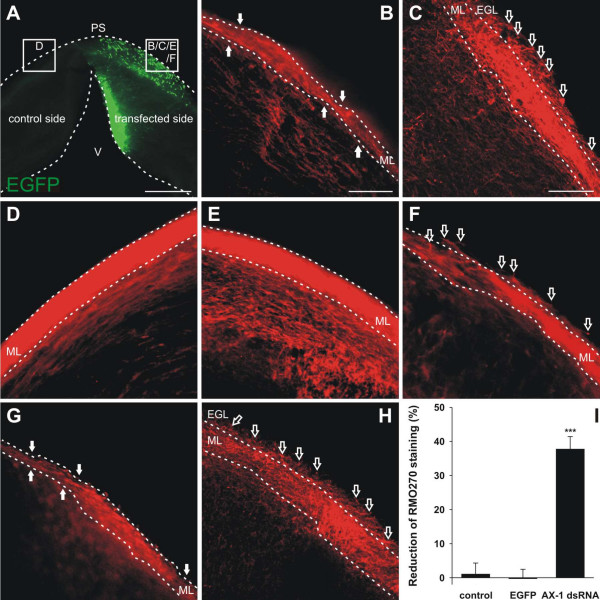
**Silencing AX-1 in the developing cerebellum induces aberrant trajectories of parallel fibers.(a-c,f) **Parallel fibers from areas outlined in (a) were analyzed in 250-μm-thick vibratome slices taken from HH35 cerebella after downregulation of AX-1 by *ex ovo *RNAi at HH34 (a-c,f). (b) In the absence of AX-1, the formation of the ML was severely disturbed in the transfected area. The width of the ML was irregular and fiber density was clearly reduced (arrows). (c) Most strikingly, axons of granule cells failed to extend in a parallel manner both with respect to each other and with respect to the pial surface (open arrows). (f) The same phenotype was obtained with a different long dsRNA derived from the AX-1 cDNA (open arrows). **(d) **On the contralateral side of the cerebellum injected and electroporated with AX-1 dsRNA the organization of the ML and the density of the parallel fibers were indistinguishable from non-injected, age-matched control embryos. **(e) **As expected, no difference between non-injected (not shown) and EGFP-injected control embryos was observed. **(g,h) **As an independent method for AX-1 perturbation, we used antibodies to block AX-1 function at the protein level. The phenotype obtained by *ex ovo *RNAi was reproduced with function-blocking antibodies. **(i) **The effect of AX-1 silencing by *ex ovo *RNAi was quantified. The relative fluorescence intensity after neurofilament staining was determined as a measure for the decrease in both ML width and parallel fiber density. On average, the staining intensity of the ML on the targeted side, that is, in the absence of AX-1, was reduced by 37.8 ± 3.6% compared to the non-affected control side (AX-1 dsRNA; n = 19 slices from 9 embryos). The staining intensities did not differ between the two halves of the cerebellum in embryos electroporated with the EGFP plasmid alone (EGFP; -0.3 ± 2.8%; n = 14 slices from 6 embryos) or in non-treated control embryos (control; 1.1 ± 3.2%; n = 10 slices from 4 embryos). ***P-values were < 0.0001 for the comparison between embryos treated with AX-1 dsRNA and EGFP-expressing control embryos, and experimental embryos versus non-treated control embryos. PS, pial surface; V, ventricle. Bar: 500 μm in (a), 100 μm in (b,d,e,g), and 50 μm in (c,f,h).

For functional analysis of AX-1 in the developing cerebellum, we injected two long dsRNA fragments that were used previously to analyze the function of AX-1 in commissural axon guidance by *in ovo *RNAi [25]. Injection of either one of the dsRNAs derived from AX-1 resulted in the same respective phenotypes in the spinal cord and in the cerebellum. In the spinal cord, loss of AX-1 caused the failure of commissural axons to cross the midline [3,4,25]. Knock down of AX-1 in the developing cerebellum at HH34 resulted in the failure of granule cell axons to extend parallel to each other and parallel to the pial surface (Figure 6). When analyzed in coronal slices at HH35, parallel fibers were found to deviate from their normal trajectory, often even invading the outermost part of the EGL, where granule cell precursors proliferate (Figure 6c,f). In control embryos injected with dsRNA derived from CD34, a gene not expressed in the cerebellum, or in control embryos injected with the EGFP plasmid alone (Figure 6e), neurofilament staining was not found in the outer EGL. The same was true for the contralateral side of experimental embryos (Figure 6d). The failure of granule cell axons to extend in a parallel manner with respect to each other and with respect to the pial surface was reflected in the irregular appearance of the ML (Figure 6b). Its width was non-uniform and the fiber density appeared strongly reduced. Because we did not observe an increase in cell death compared to control embryos (Figure 2) we took the apparent decrease in fiber number in the developing ML as further evidence for the aberrant growth of parallel fibers. Because it was impossible to count the number of aberrant fibers individually, we measured the intensity of neurofilament staining in the developing ML. As the width of the layer varies considerably depending on the anteroposterior position in the cerebellum, we measured the staining intensity as a ratio between the targeted and the contralateral side. The comparison of neurofilament staining intensity between the experimental and the control sides indicated a 38% decrease in AX-1 dsRNA-treated embryos (n = 19 slices from 9 embryos) but no changes in EGFP-treated embryos (n = 14 slices from 6 embryos) or untreated embryos (n = 10 slices from 4 embryos) (Figure 6i).

These results were reproduced qualitatively in embryos treated with function-blocking anti-AX-1 antibodies (Figure 6g,h). Thus, perturbation of AX-1 function either by *ex ovo *RNAi or by injection of function-blocking antibodies resulted in aberrant growth of granule cell axons.

### Knock down of AX-1 interferes with guidance but not growth of parallel fibers

**Figure 7:**
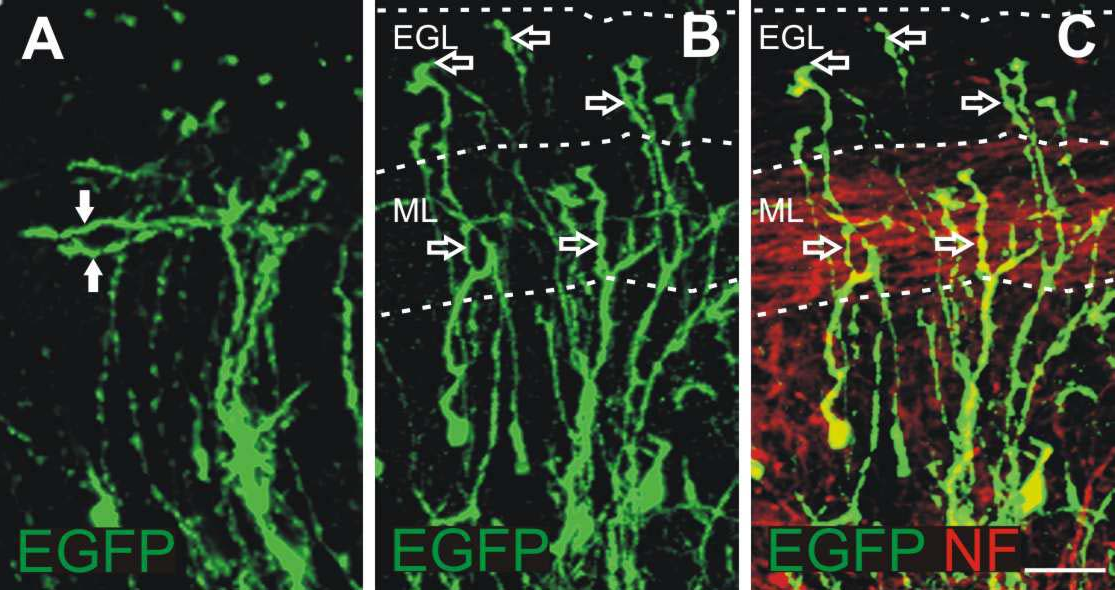
**Guidance but not growth of granule cell axons is affected in the absence of AX-1 *in vivo*.(a) **Detailed analyses of coronal slices taken from control-injected embryos at HH35 revealed correctly formed parallel fibers expressing EGFP (arrows). **(b,c) **In contrast, EGFP-expressing granule cells in embryos injected and electroporated with AX-1 dsRNA and the EGFP plasmid did not extend their axons parallel to, but rather towards, the pial surface (open arrows). (c) Overlay with a neurofilament staining outlines non-electroporated parallel fibers in the developing ML. Bar: 25 μm.

Loss of AX-1 *in vivo *did not prevent the formation of granule cell axons, but interfered with their orientation (Figure 7). Previous *in vitro *studies did not detect an effect of TAG-1/AX-1 on mouse granule cell axon extension [41]. Consistent with these results, we found that blocking AX-1 function *in vivo *did not interfere with axon extension but with the direction of their growth. The comparison of granule cells in slices taken from control-injected embryos and embryos lacking AX-1 at high magnification revealed normal T-shaped axons in controls at HH35 (Figure 7a) but aberrant growth of axons toward the pial surface in experimental embryos (Figure 7b,c).

### The aberrant growth of fibers is not caused by changes in granule cell development and maturation

**Figure 8:**
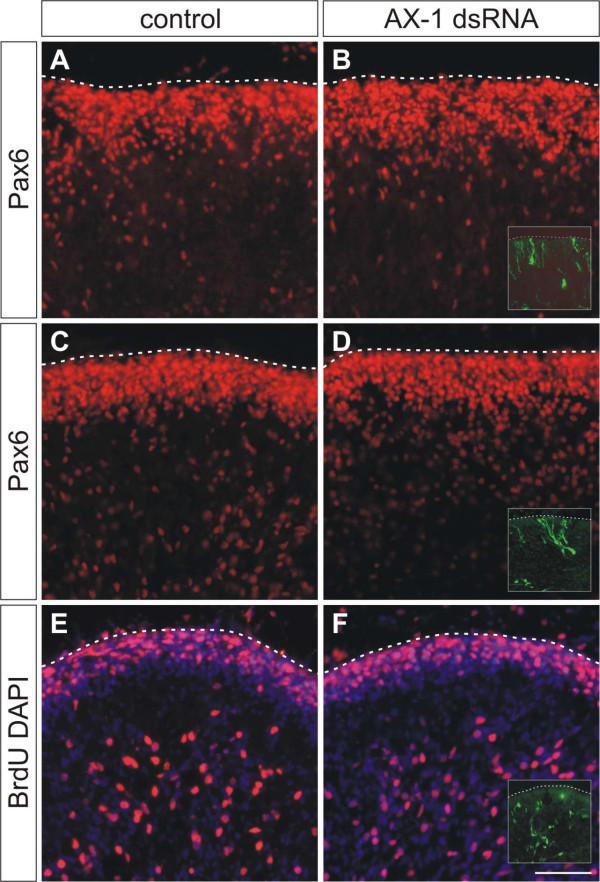
**Downregulation of AX-1 does not interfere with granule cell development or migration.** Perturbation of AX-1 expression did not interfere with granule cell migration from the EGL through the ML. **(a-d) **No change in the distribution of Pax6-positive granule cells was detectable between untreated control embryos at HH35 (a) or HH38 (c) and embryos injected and electroporated with dsRNA derived from AX-1 at HH35 (b) or HH38 (d). **(e,f) **As expected, downregulation of AX-1 by *ex ovo *RNAi at HH34 did not interfere with proliferation of granule cell precursors, as AX-1 is expressed only after granule cells become postmitotic. Sagittal sections labeled with BrdU and counterstained with DAPI taken from control embryos (e) and embryos lacking AX-1 (f) at HH38 did not differ. Inserts in (b,d,f) show adjacent sections stained for EGFP to demonstrate that sections were taken from the electroporated area of the cerebellum. Bar: 50 μm.

To rule out an effect of aberrant granule cell development on parallel fiber formation, we analyzed the migration of granule cells from the EGL through the developing ML. For this purpose we used Pax6 as a marker for granule cell somas [42] at HH35 (Figure 8a,b) and HH38 (Figure 8c,d). Downregulation of AX-1 did not interfere with granule cell migration (Figure 8b,d) compared to untreated controls (Figure 8a,c). Furthermore, the lack of AX-1 did not interfere with granule cell maturation. In particular, the number and position of proliferating granule cell precursors in the outer EGL was indistinguishable between control embryos (Figure 8e) and experimental embryos (Figure 8f). Thus, lack of AX-1 did not interfere with granule cell proliferation and migration.

### AX-1 is required for parallel fiber formation but not for neurite extension *in vitro*

**Figure 9:**
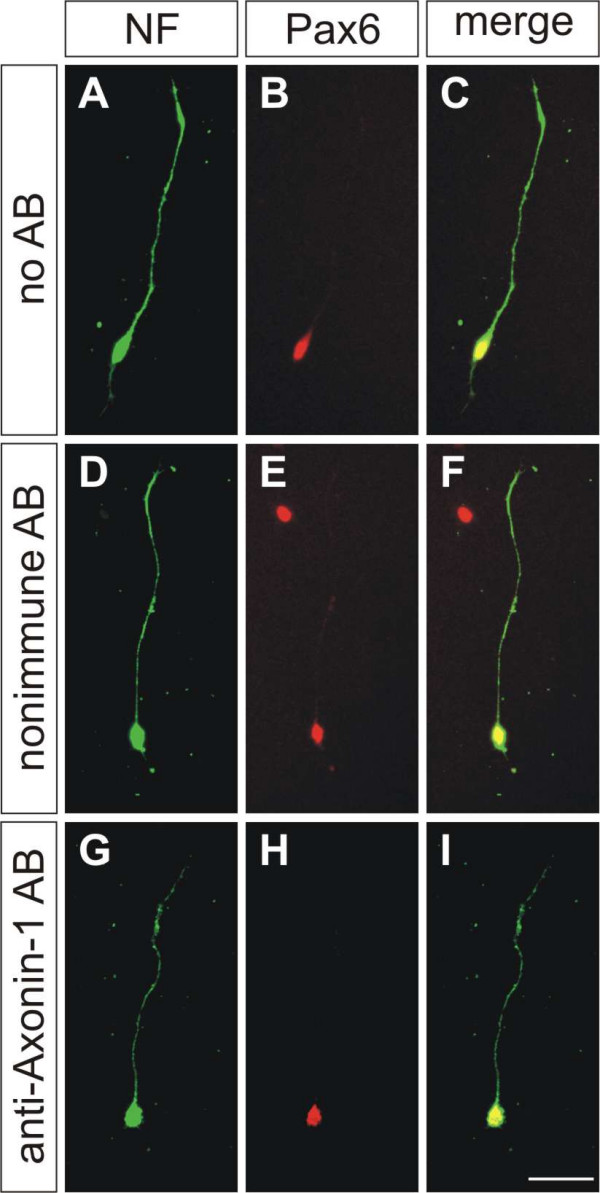
**AX-1 is not required for neurite extension on laminin.(a-i) **Granule cells were plated on laminin in the absence of antibodies (AB) (a-c), in the presence of non-immune antibodies (d-f), and in the presence of goat anti-AX-1 antibodies (g-i). Neurites were stained with a rabbit anti-neurofilament (NF) antibody (a,d,g). Granule cells were identified by Pax6 (b,e,h). Merged images are shown in (c,f,i). Neurite morphologies and lengths did not differ under these conditions. Values were 101.0 ± 7.3 μm (n = 27) in the absence of antibodies, 101.1 ± 9.7 μm (n = 23) in the presence of non-immune, and 94.5 ± 7.6 μm (n = 31) in the presence of anti-AX-1 antibodies. Bar: 25 μm.

To address the mechanism underlying the observed failure of granule cell axons to extend in a parallel manner to each other and to the pial surface, we investigated the role of AX-1 in neurite outgrowth promotion *in vitro*. When laminin was used as a substrate, granule cell axons grew almost exclusively on or in close contact with glia cells. Blocking AX-1 function did not interfere with this behavior nor change neurite length (Figure 9). Neurite length was 101.0 ± 7.3 μm for granule cells grown without antibodies added to the medium, 101 ± 9.7 μm in the presence of non-immune goat IgG, and 94.5 ± 7.6 μm in the presence of goat anti-AX-1 IgG.

**Figure 10:**
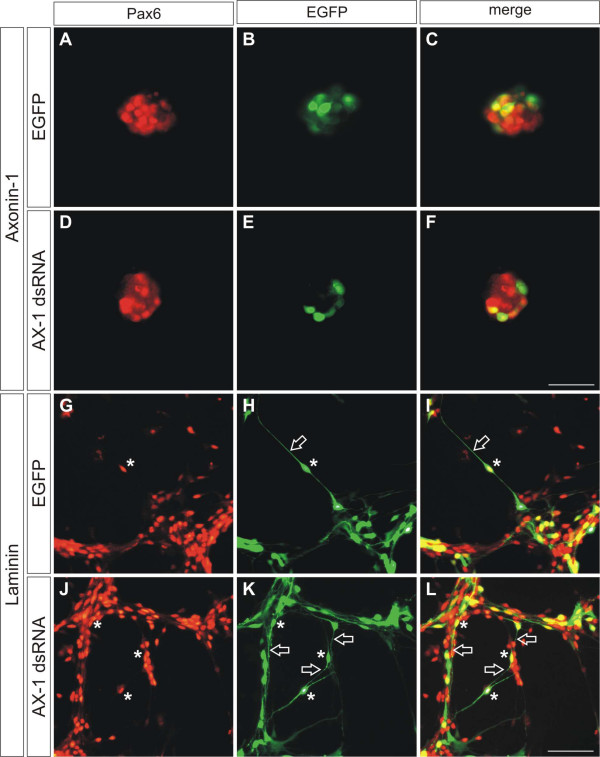
**AX-1 does not promote outgrowth of granule cell axons.(a-f) **When granule cells were cultured on AX-1 as a substrate they failed to extend neurites, no matter whether they expressed AX-1 (a-c) or not (d-f). Granule cells were electroporated with dsRNA derived from AX-1 and the EGFP plasmid *in vitro*, before they were cultured on AX-1 substratum (d-f) and compared with cells electroporated with the EGFP plasmid only (a-c). **(g-l) **On laminin substratum, both granule cells expressing AX-1 (g-i) and those lacking AX-1 (j-l) extended axons. Granule cells were identified by Pax6 expression (a,d,g,j). Electroporated cells expressed EGFP (b,e,h,k). Merged images are shown in (c,f,i,l). Asterisks indicate electroporated granule cells identified by EGFP and Pax6 expression. Open arrows label axons from Pax6-positive neurons that also express EGFP. Bars: 25 μm in (a-f) and 50 μm in (g-l).

Further confirmation for the absence of an effect of AX-1 on granule cell axon outgrowth was found when we coated culture dishes with purified AX-1. As described earlier [43,44], AX-1 supported axon outgrowth and the formation of characteristic, large growth cones from dorsal root ganglion neurons. However, under the same conditions, we did not detect neurite outgrowth from granule cells (Figure 10), confirming previous results obtained with mouse granule cells and TAG-1 [41]. In agreement with our previous observations of granule cells cultured in the presence of function-blocking antibodies (Figure 9), they extended neurites on laminin, no matter whether they expressed AX-1 or not, that is, when AX-1 was downregulated by *in vitro *electroporation before plating (Figure 10g–l). In contrast, no axons were found on AX-1 used as a substrate (Figure 10a–f). Therefore, we concluded that AX-1 was required for axon guidance but not for axon growth, in agreement with earlier observations made for commissural neurons from the dorsolateral spinal cord [45].

## Discussion

The chicken embryo was rediscovered as a modern model organism for developmental studies due to the use of electroporation for efficient gene transfer *in vivo *[33,35,46-48] (reviewed in [26,27]). With this method, gain-of-function studies by spatially and temporally controlled expression of a target gene became possible [33,49-53]. However, loss-of-function phenotypes still depended on the availability of dominant-negative forms of candidate proteins. Therefore, the combination of *in ovo *electroporation and RNAi that allowed for temporally and spatially controlled gene silencing was an important step forward [25,31,54]. Functional studies by *in ovo *RNAi using long dsRNA, short interfering RNAs, or short hairpin RNAs have been carried out in various parts of the central nervous system but also in other embryonic tissues [25,31,54-57]. At later stages of development, the accessibility of the growing embryo through the small window in the eggshell is restricted and, therefore, the time window for *in ovo *electroporation closes at around E5.

In this study, we show that *ex ovo *electroporation and RNAi can be used for functional gene analysis at late developmental stages in the developing cerebellum. In the chick, the formation of the distinct cerebellar layers starts around HH34 (E8). The cerebellum represents an ideal system to investigate the multiple processes contributing to central nervous system development, including cell proliferation [58], differentiation [59], migration [60,61], axon growth and guidance [40], as well as synaptogenesis [62].

Luo and Redies [33] demonstrated that *ex ovo *electroporation can be used to transfect Purkinje cells. Using a tungsten needle as cathode, Purkinje cell progenitors were successfully electroporated after injection of a GFP plasmid into the ventricular system at E3.5. However, temporally and spatially controlled transfection of different cerebellar cell types was not possible at these early stages [33,35]. Furthermore, electroporation of antisense cDNA to induce a loss-of-function phenotype at E3.5 was not sufficient for functional readouts at later stages of cerebellar development (E11.5) [63]. Due to ongoing cell proliferation, transfected antisense cDNAs were diluted extensively, resulting in inefficient downregulation of the target gene. An additional problem with the early electroporation of antisense nucleotides, but also dsRNA for RNAi, may occur if a candidate gene exerts multiple functions at different stages of development. Early disruption of gene function would prevent any further functional analysis at later developmental stages. Therefore, our focus was to design appropriate conditions to extend the applicability of RNAi to older stages of embryonic development. With this protocol, AX-1 in granule cells of the EGL could efficiently be downregulated (Figure 3).

Despite the fact that some genes involved in cerebellar specification have been identified [13], the molecular basis of cerebellar development is still poorly understood [9,64]. A number of cell adhesion molecules of the immunoglobulin superfamily are expressed in granule cells, including NgCAM/L1 [38], NrCAM [38], Contactin/F11/F3 [40,65], and AX-1/TAG-1 [6-8]. *In vitro *studies indicated an inhibitory role of Contactin/F3 in parallel fiber outgrowth [66]. *In vivo*, parallel fibers extended in the absence of Contactin/F3 but their growth and contact with dendrites of Purkinje cells was aberrant [40]. Only later extending parallel fibers were affected in contactin/F3 knockout mice, growth of early extending fibers was normal, consistent with the late onset of contactin/F3 expression in granule cells compared to TAG-1/AX-1 [8,39]. A forced early expression of Contactin/F3 under the TAG-1 promoter interfered with the correct formation of the ML [39]. Reduced cerebellar size and defects in foliation were found in mice lacking both L1/NgCAM and NrCAM [38]. The defect was the result of a lack of granule cell survival rather than an effect on parallel fiber growth. Only very subtle cerebellar phenotypes were found in mice lacking only one of the two genes [38]. Mice lacking TAG-1 were shown to have a migration defect of a subset of pre-cerebellar neurons in the superficial migratory stream, but no cerebellar phenotype was reported in these mice [67,68]. The absence of an effect on granule cells was unexpected based on the expression of TAG-1/AX-1 in postmitotic granule cells at the time of parallel fiber extension [68]. Similarly, no effect on commissural axons was found in the spinal cord of the *Tag-1 *knockout mouse [68], in contrast to the chicken embryo where the perturbation of AX-1 function resulted in the failure of commissural axons to cross the midline [3,25]. The most likely explanation for this discrepancy is the technique used to block TAG-1/AX-1 function. While TAG-1 is permanently and ubiquitously deleted in the knock-out mouse, AX-1 function was blocked acutely during a specific time window of interest in our *ex ovo *RNAi approach ([25]; this study) or by the injection of function-blocking antibodies [3,4]. Therefore, redundancy or compensatory mechanisms that are generally thought to explain the absence of phenotypes in knock-out mice [38,67,68] would not be present in the 'acute' knock down of AX-1 in chicken embryos. Such a scenario would also explain the discrepancy between the *NrCAM *knockout mouse [69] and our previous reports on the effect of acute perturbation of NrCAM function by injection of function-blocking antibodies [3,5] and *in ovo *RNAi [25] on axon guidance in the spinal cord. Thus, despite the fact that acute knock down of a gene of interest by RNAi is not removing all of the gene product, because preexisting protein is not removed and only a subset of the cells in the target area are transfected by electroporation, this approach may be more sensitive for the analysis of gene function during embryonic development compared to generic knock-out approaches that are the method of choice when gene function in the adult nervous system is of interest.

Downregulation of AX-1 in the developing cerebellum by *ex ovo *RNAi specifically interfered with the formation of parallel fibers. This defect was not caused by inhibition of axon extension, as we found no effect of AX-1 on neurite length *in vitro*. Furthermore, our analyses of cerebellar slices taken from embryos lacking AX-1 are consistent with an effect of AX-1 on guidance but not on growth of granule cell axons. Granule cells still extended axons after downregulation of AX-1, although their orientation was aberrant (Figure 7).

These results are consistent with earlier observations made with commissural neurons from the dorsolateral spinal cord *in vitro *and *in vivo *[3,45]. AX-1 was required for the choice of commissural axons between different substrates that supported axon extension, but had no effect on axon growth *in vitro*. The same conclusion was drawn from *in vivo *studies where the absence of AX-1 resulted in the failure of commissural axons to cross the midline but did not affect neurite extension that was shown to depend on NgCAM and NrCAM. The effect of AX-1 on granule cell axon guidance is most likely due to a homophilic AX-1/AX-1 interaction [70,71], as the granule cells that extend axons early do not express any of the known binding partners of AX-1, such as NgCAM (Figure 4b), NrCAM (Figure 4e), or Contactin/F11 (Figure 4h; [39]). Expression of those molecules is found only in older parallel fibers in the developing ML. Furthermore, a homophilic interaction of AX-1 is consistent with all our *in vitro *and *in vivo *results.

## Conclusion

AX-1 is expressed by granule cells as soon as they become postmitotic and is required for the parallel arrangement of their axons but is not required for neurite extension. This is similar to the situation found for commissural axons of the spinal cord. They require AX-1 for pathfinding but not for elongation. In contrast, AX-1 was found to be required for guidance of sensory DRG axons *in vivo *[5] and to promote their outgrowth *in vitro *[43,44]. Taken together, these results demonstrate that AX-1 acts in a context-dependent manner either as an axon guidance cue without affecting neurite outgrowth, or as a molecule promoting neurite outgrowth. In the cerebellum, AX-1 expressed by postmitotic granule cells is required for the navigation of parallel fibers in the developing ML.

## Materials and Methods

### *Ex ovo *culture for chicken embryos

Fertilized Hisex eggs were obtained from a local hatchery and pre-incubated for 2.5 days at 38.5°C with a humidity of at least 45%. After the egg had been positioned on the side for at least 20 minutes to allow for the embryo to float on top of the yolk, it was wiped with 70% ethanol, and cracked on a sharp edge. The whole egg content was transferred carefully into a domed dish with a diameter of 80 mm and a depth of 40 mm. These dishes were produced for the food industry from oriented polystyrene (OPS; Bellaplast, Altstaetten, Switzerland; Additional file 1). To minimize evaporation, the dish was covered with a lid. The *ex ovo *cultures were kept in an egg incubator at 38.5°C (Heraeus B12, Therrmo Fisher Scientific, Wohlen, Switzerland). To evaluate the survival rate at different stages of development, we opened the incubator once a day to count survivors (Additional file 1d). However, for routine experiments and best survival rate the incubator was not opened for the first 6 days of *ex ovo *culturing. Under these conditions the survival rate was much better (on average 62% at E8). Compared to the *ex ovo *culturing method described by Luo and Redies [35], the use of a domed dish was advantageous. We had better survival for a longer period of time in the domed polystyrene dishes. Contaminations were very rare (much less than 1%), although we did not work in a laminar flow hood.

### Synthesis of dsRNA

For *in vitro *transcription, 2 μg of the linearized (cut with *Bam*HI and *Sac*I; Roche, Basel, Switzerland) and purified plasmids encoding AX-1 (either nucleotides 28–1,592 or nucleotides 1,620–2,298 of the full-length cDNA; see [25] for details) were mixed with 0.8 μl 100 mM rNTPs (25 mM each; Roche), 0.5 μl RNasin (Promega, Duebendorf, Switzerland), 2 μl Sp6 or T7 RNA polymerase (15 U/μl; Promega), and 2 μl 100 mM DTT in transcription buffer (final volume 20 μl). After 4 h at 37°C, the DNA template was removed from the *in vitro *transcription mixture by digestion with 20 units RNase-free DNaseI (Roche) for 1 h at 37°C. For dsRNA derived from CD34, nucleotides 1,640–2,417 of the full-length cDNA (XM_417984) were used. The plasmid was linearized with *Not*I (New England Biolabs, Ipswich, MA, USA) and *Eco*RI (Roche) and single-stranded RNA (ssRNA) was prepared using T3 and T7 RNA polymerases. The synthesized ssRNA was purified by sequential extraction with acidic phenol-chloroform (25:24:1 vol/vol/vol phenol/chloroform/isoamyl alcohol) and chloroform/isoamylalcohol (24:1). After precipitation with 100% ethanol, the ssRNA was washed with 70% ethanol, and dissolved in 20 μl phosphate-buffered saline (PBS). To produce dsRNA, equal nanogram amounts of antisense and sense ssRNAs were mixed, heated for five minutes at 95°C, and then slowly cooled down to room temperature by switching off the heating block. For quality control, 1 μl samples were taken after each step and analyzed by gel electrophoresis. The dsRNA was stored at -80°C until further use.

### *Ex ovo *injection and electroporation

Injections and electroporations were performed at E8. The embryos were staged according to Hamburger and Hamilton [36]. To have direct access to the embryo, a small hole of 3–4 mm diameter was cut into the extraembryonic membranes above the eye. For positioning and stabilization of the head during injection and subsequent electroporation, we used a hook prepared from a spatula (Figure 1a). Approximately 1 μl of the nucleic acid mixture consisting of a plasmid encoding EGFP under the control of the β-actin promoter (100 ng/μl), dsRNA derived from AX-1 (500 ng/μl), and 0.04% (vol/vol) Trypan Blue (Invitrogen, Carlsbad, CA, USA) dissolved in sterile PBS were injected into the cerebellum using a borosilicate glass capillary with a tip diameter of 5 μm (Figure 1a; World Precision Instruments, Berlin, Germany). Depending on the depth of the injection, different cerebellar layers could be targeted. To get transfection of all cerebellar layers, the glass capillary was first inserted into the ventricular system and injection pressure was maintained during retraction. Before electroporation a few drops of sterile PBS were added to the embryo. For the electroporation a platelet electrode of 7 mm diameter (Tweezertrodes Model #520, BTX Instrument Division, Harvard Apparatus, Holliston, MA, USA) was placed collaterally to the head of the embryo (Figure 1b). Six pulses of 40 V and 99 ms duration with one second interpulse intervals were applied using a square wave electroporator (ECM830, BTX). For the present study, we selected only embryos that were successfully electroporated in the EGL and the developing ML for further analysis. Slices and sections from experimental embryos were compared with slices/sections taken from the same position of a control cerebellum with respect to both the anteroposterior and the mediolateral axis. As controls we used CD34 dsRNA injected, EGFP plasmid injected, and untreated control embryos, respectively.

For injections of function-blocking anti-AX-1 antibodies, approximately 1 μl of the antibody solution (10 mg/ml with 0.04% Trypan Blue in PBS) was injected into the cerebellum three times every 12 h.

### Tissue preparation

One to four days after electroporation the embryos were sacrificed. The brain was removed and analyzed for EGFP expression using a fluorescence stereomicroscope (SZX12, Olympus). After fixation for 2 h at room temperature in 4% paraformaldehyde in PBS, the brain tissue was rinsed in PBS and transferred to 25% sucrose in 0.1 M sodium phosphate buffer, pH 7.4, for cryoprotection. In this study, 30-μm-thick sagittal cryostat sections and 250-μm-thick coronal vibratome slices were used. For the preparation of cryostat sections, the brains were embedded with OCT Tissue-Tek in Peel-a-Way^® ^disposable embedding molds (Polysciences, Eppelheim, Germany), frozen in isopentane on dry ice, and cut on a cryostat (Leica CM1850). The sections were collected on SuperFrost^®^Plus microscope slides. For the preparation of 250-μm-thick vibratome slices, the brain was embedded in 6.5% ultra low-melting agarose (Type IX, Sigma, Buchs, Switzerland) and cut with an OTS-300-04 oscillating tissue slicer (Electron Microscopy Sciences, Hatfield, PA, USA). The slices were collected in PBS and the remaining agarose was removed before staining.

### Immunohistochemistry

Immunostaining of cryostat sections was done essentially as described earlier [5]. We used the following primary antibodies: rabbit anti-GFP (1:250; Abcam, Cambridge, UK), FITC-labeled goat anti-GFP 1:400; Rockland, Gilbertsville, PA, USA), anti-Calbindin (1:1000; Swant, Bellinzona, Switzerland), both rabbit and goat anti-AX-1 (1:1,000) [3,25], goat anti-NgCAM, anti-NrCAM (both 1:1,000), rabbit anti-Contactin/F11 (1:1,000) [5], mouse anti-neurofilament (RMO270; 1:1,500; Zymed/Invitrogen, Carlsbad, CA, USA), rabbit anti-neurofilament (1:250; Chemicon/Millipore, Billerica, MA, USA), and the monoclonal antibodies H5, recognizing Vimentin (supernatant), and Pax6 (2 μg/ml; both obtained from the Developmental Studies Hybridoma Bank, University of Iowa, Iowa City, IA, USA). All antibodies were diluted in blocking buffer (10% fetal calf serum in PBS). Incubation was overnight at 4°C for primary antibodies and 90 minutes at room temperature for secondary antibodies. Secondary antibodies were: goat anti-rabbit-Alexa488 and donkey anti-goat-Alexa488 (1:250; Molecular Probes/Invitrogen), goat anti-mouse-Cy3 (1:250), donkey anti-rabbit-Cy3 (1:200), or donkey anti-goat-Cy3 (1:250; all Jackson ImmunoResearch Laboratories, Newmarket, Suffolk, UK). For staining of vibratome slices, essentially the same protocol was used, although incubation times were extended [72]. Slices were mounted in sterile PBS between two coverslips sealed with high vacuum grease (Dow Corning, Wiesbaden, Germany).

### **Bromodeoxyuridine labeling**

Embryos were injected with dsRNA derived from AX-1 and the EGFP plasmid, or with the EGFP plasmid alone, and electroporated at HH34. After 4 days (HH38), 200 μl 50 mM bromodeoxyuridine (BrdU) in H_2_O were pipetted onto the chorioallantois. After 3 h the embryos were sacrificed, and the brains were dissected and prepared for cryostat sections as described above. For visualization of the incorporated BrdU, the sections were incubated in 50% formamide, 0.3 M NaCl in 0.03 M tri-sodium citrate buffer, pH 7.0, for 1–2 h at 65°C, rinsed twice with 0.3 M NaCl in 0.03 M tri-sodium citrate buffer for 15 minutes, followed by incubation in 2 N HCl for 30 minutes at 37°C. Sections were rinsed in 0.1 M borate buffer (pH 8.5) for 10 minutes at room temperature, followed by PBS (six changes). BrdU was detected using the mouse anti-BrdU antibody from Sigma (1:200) using the protocol detailed above. Sections were counterstained with DAPI (5 μg/ml in PBS) for 20 minutes at room temperature.

### TUNEL assay

Apoptosis was compared between embryos taken directly from the egg, control embryos that were injected and electroporated with the EGFP plasmid alone, or experimental embryos injected with dsRNA derived from AX-1 (together with the EGFP plasmid). As a positive control, sections were treated with DNase I (300 U/ml; Roche) for 10 minutes at room temperature. To detect apoptotic cells, the ApoAlert DNA Fragmentation Assay Kit (Clontech, Mountain View, CA, USA) was used according to the manufacturer's instructions. The fragmented, fluorescein-labeled DNA was detected with an alkaline phosphatase-conjugated sheep anti-FITC antibody (1:1,000; Roche) dissolved in 10% fetal calf serum/PBS. The alkaline phosphatase reaction was carried out as described earlier [73].

### Quantification

Fluorescence intensities after AX-1 or neurofilament staining were measured with the analySIS Five software from Olympus Soft Imaging System (Muenster, Germany). A decrease of the protein level was calculated from the relative fluorescence intensities measured in the ML of the transfected versus non-transfected areas. The staining intensity was then compared between embryos injected with dsRNA derived from AX-1, embryos injected with the EGFP plasmid only, and untreated controls. For statistical analyses ANOVA with Bonferroni correction was used. Values are given as mean ± standard error of the mean. *P*-values were < 0.0001.

### *In vitro *assays

Granule cells were collected by microdissection of the cerebellum at HH36. The EGL was separated from coronal slices by cutting through the ML. The tissue was collected in ice-cold HBSS (Hank's Balanced Salt Solution). After trypsinization and trituration, cells were suspended in MEM containing 10% fetal calf serum and penicilin/streptomycin, and plated at a density of 50,000 cells per well of an 8-well LabTek slide. Slides were coated with 20 μg/ml laminin or 50 μg/ml AX-1 essentially as described in [44] except that slides coated with laminin were precoated with 100 μg/ml poly-lysine. After 48 h, cells were fixed in 4% paraformaldehyde for 1 h at room temperature. Cell cultures contained granule cells and Bergmann glia cells when coated on laminin as determined by Pax6 and GFAP staining (not shown). On AX-1 substratum glia cells were not able to spread. Neurite lengths were measured and are representative for four different cultures. Antibodies were added to the culture medium at a concentration of 5 μg/ml.

### *In vitro *electroporation

Granule cells suspended in culture medium (see above), 40 ng/μl EGFP plasmid, and 5 ng/μl dsRNA derived from AX-1 were electroporated using the BTX Safety Stand 630B and matching cuvettes connected to the BTX Electro Square Porator ECM830. After electroporation with 1 pulse of 5 ms duration at 112 V cells were kept at 37°C for 5 minutes before plating. When cells were subject to electroporation before culturing they were plated at a density of 800,000 per well.

## Abbreviations

AX, axonin; dsRNA, double-stranded RNA; BrdU, bromodeoxyuridine; E, embryonic day; EGFP, enhanced green fluorescent protein; EGL, external germinal layer; HH, Hamburger and Hamilton stage; ML, molecular layer; PBS, phosphate-buffered saline; RNAi, RNA interference; Shh, Sonic hedgehog; ssRNA, single-stranded RNA.

## Competing interests

The author(s) declare that they have no competing interests.

## Authors' contributions

TB carried out the experiments, participated in drafting the manuscript and prepared the figures. ES conceived the study, participated in its design and wrote the manuscript. Both authors read and approved the manuscript.

## Supplementary Material

Additional file 1 Chicken embryos can be cultured *ex ovo *to make them easily accessible for manipulations throughout embryonic development. (a) After two days of incubation at 38.5°C the whole egg content was carefully transferred into a domed dish. After 6 days of *ex ovo *culturing the embryos reached HH34/E8 (b), the time point when injections and electroporations were performed in this study. The chicken embryo can be kept alive throughout embryonic development. Due to an increasing number of blood vessels in the extraembryonic membranes at later developmental stages (as seen at E18 in (c)), injections and electroporations become more difficult, however. Depending on the time of transfer the survival rate varied slightly but not significantly. (d) When embryos were transferred after two days (black dots), the survival rate of the embryos decreased more slowly during the first eight days compared to embryos that were transferred after 3 days (white dots). In both cases the survival rates stabilized for the following days (after E8). Routinely, we did not take *ex ovo *cultures out of the incubator before E8, the time point of injection. This resulted in a markedly higher survival rate of 62% at E8 (red dot). The age of the embryos is indicated in days and corresponding developmental stages according to Hamburger and Hamilton [36]. Bar: 1 cm. Click here for file

Additional file 2 Culturing embryos *ex ovo *does not disturb cerebellar development. The morphology of the cerebellum at HH38, revealed by methylene blue staining, was indistinguishable between embryos developing in the egg (a) and embryos that had been in a dish for 10 days (b). HH38 is the oldest developmental stage used in this study. Similarly, no differences between embryos developing in the egg (c,e) and embryos developing in a domed polystyrene dish (d,f) were found at HH38 when more specific criteria were used, like migration of Purkinje cells and formation of the characteristic layer in the periphery of the lobes (c,d) or AX-1 staining of parallel fibers in the ML (e,f). Purkinje cells were stained with an anti-Calbindin antibody (c,d). Therefore, differences in development between experimental and control embryos were not caused by culturing embryos in a dish. Bar: 500 μm in a,b; 100 μm in c-f. Click here for file
