# Supplementary figures and images for: Axonin-1/TAG-1 is required for pathfinding of granule cell axons in the developing cerebellum
Source: Neural Dev. 2008 Mar 17;3:7. doi: 10.1186/1749-8104-3-7 (PMC2322981; doi:10.1186/1749-8104-3-7)

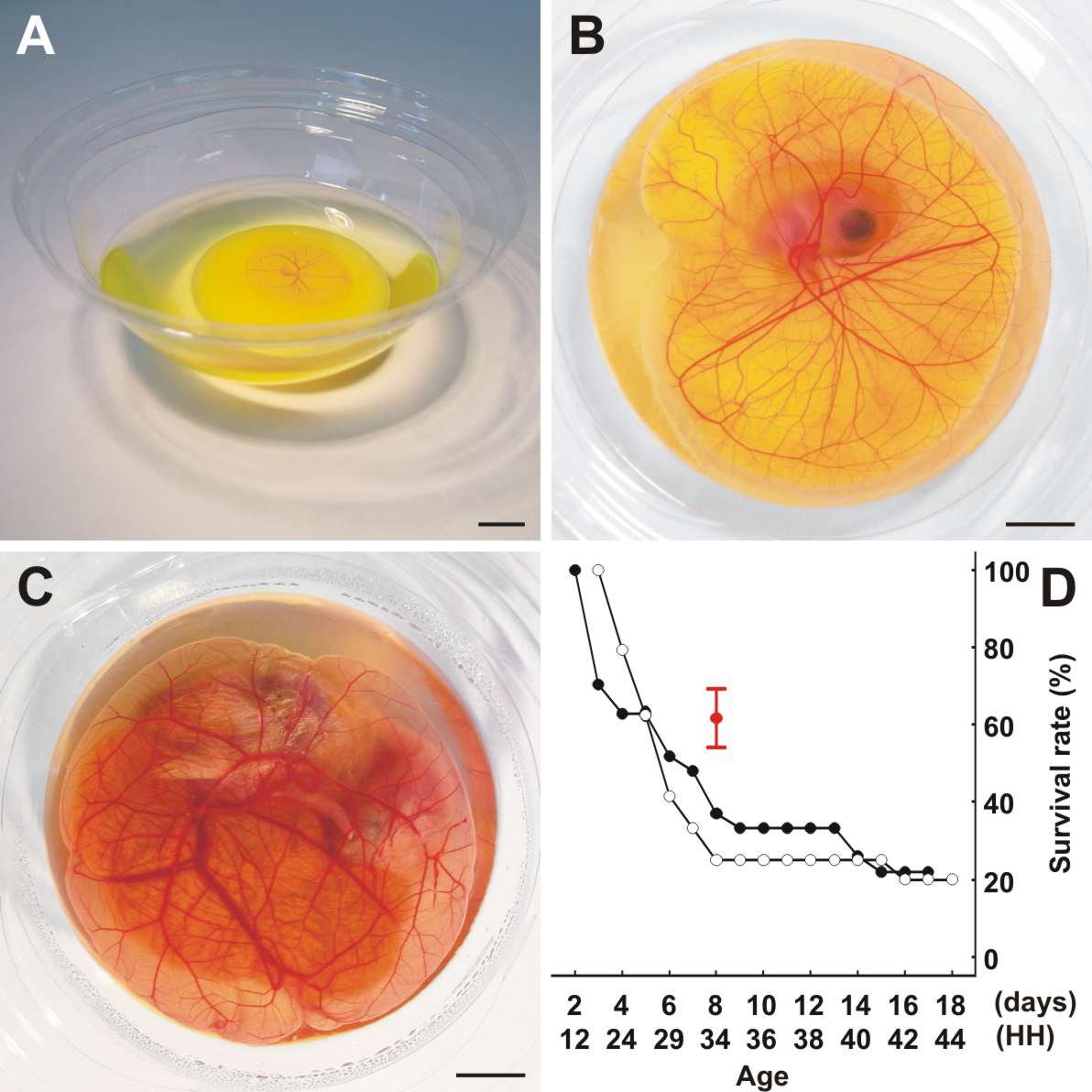

Supplement: Additional file 1 — Chicken embryos can be cultured ex ovo to make them easily accessible for manipulations throughout embryonic development. (a) After two days of incubation at 38.5°C the whole egg content was carefully transferred into a domed dish. After 6 days of ex ovo culturing the embryos reached HH34/E8 (b), the time point when injections and electroporations were performed in this study. The chicken embryo can be kept alive throughout embryonic development. Due to an increasing number of blood vessels in the extraembryonic membranes at later developmental stages (as seen at E18 in (c)), injections and electroporations become more difficult, however. Depending on the time of transfer the survival rate varied slightly but not significantly. (d) When embryos were transferred after two days (black dots), the survival rate of the embryos decreased more slowly during the first eight days compared to embryos that were transferred after 3 days (white dots). In both cases the survival rates stabilized for the following days (after E8). Routinely, we did not take ex ovo cultures out of the incubator before E8, the time point of injection. This resulted in a markedly higher survival rate of 62% at E8 (red dot). The age of the embryos is indicated in days and corresponding developmental stages according to Hamburger and Hamilton [36]. Bar: 1 cm. [file 1749-8104-3-7-S1.jpeg]

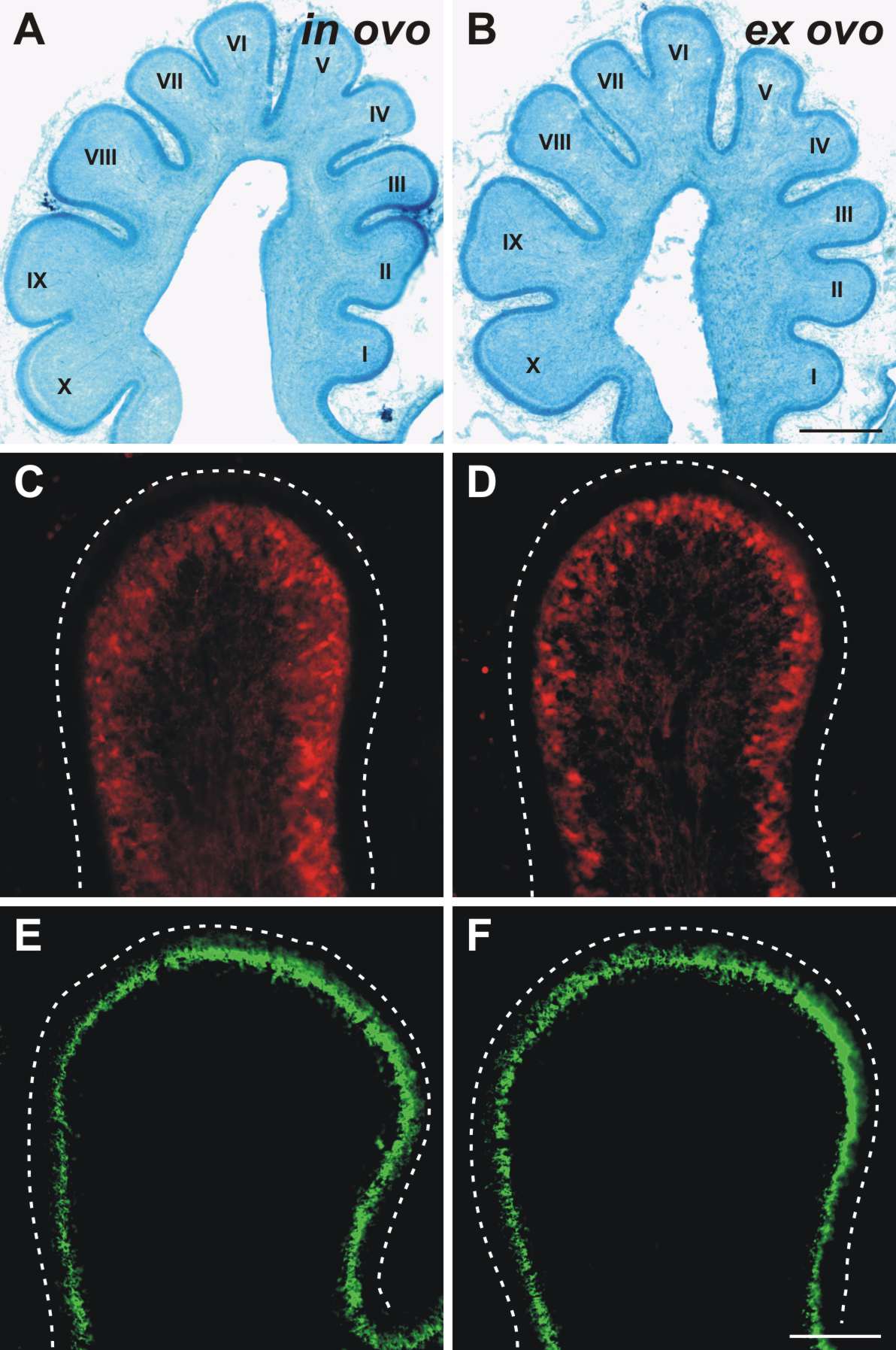

Supplement: Additional file 2 — Culturing embryos ex ovo does not disturb cerebellar development. The morphology of the cerebellum at HH38, revealed by methylene blue staining, was indistinguishable between embryos developing in the egg (a) and embryos that had been in a dish for 10 days (b). HH38 is the oldest developmental stage used in this study. Similarly, no differences between embryos developing in the egg (c,e) and embryos developing in a domed polystyrene dish (d,f) were found at HH38 when more specific criteria were used, like migration of Purkinje cells and formation of the characteristic layer in the periphery of the lobes (c,d) or AX-1 staining of parallel fibers in the ML (e,f). Purkinje cells were stained with an anti-Calbindin antibody (c,d). Therefore, differences in development between experimental and control embryos were not caused by culturing embryos in a dish. Bar: 500 μm in a,b; 100 μm in c-f. [file 1749-8104-3-7-S2.jpeg]
